# Multi-Component Passivators Regulate Heavy Metal Accumulation in Paddy Soil and Rice: A Three-Site Field Experiment in South China

**DOI:** 10.3390/toxics10050259

**Published:** 2022-05-18

**Authors:** Shouping Zhao, Xuezhu Ye, De Chen, Qi Zhang, Wendan Xiao, Shaofu Wu, Jing Hu, Na Gao, Miaojie Huang

**Affiliations:** 1 State Key Laboratory for Managing Biotic and Chemical Threats to the Quality and Safety of Agro-Products, Key Laboratory of Information Traceability for Agricultural Products, Institute of Agro-Product Safety and Nutrition, Zhejiang Academy of Agricultural Sciences, Hangzhou 310021, China; zhaosp@zaas.ac.cn (S.Z.); chend@zaas.ac.cn (D.C.); zhangq@zaas.ac.cn (Q.Z.); xiaowd@zaas.ac.cn (W.X.); hujing987@hotmail.com (J.H.); gaonagaona987@hotmail.com (N.G.); huangmj987@hotmail.com (M.H.); 2 Shaoxing Grain and Oil Crop Technology Extension Center, Shaoxing Agricultural Bureau, Shaoxing 312000, China; sf_wu@163.com

**Keywords:** biochar, lime, sepiolite, cadmium, soil enzyme, microbial carbon

## Abstract

To fulfill sustainability principles, a three-site field experiment was conducted to screen suitably mixed passivators from lime + biochar (L + C, 9000 kgha^−1^ with a rate of 1:1) and lime + biochar + sepiolite (L + C + S, 9000 kg ha^−1^ with a rate of 1:1:1), in Yuecheng (YC), Zhuji (ZJ), and Fuyang (FY), where there are typical contaminated soils, in South China. Treated with passivators in soil, DTPA-extractable Cd, Cr and Pb in soil were decreased by 9.87–26.3%, 37.2–67.5%, and 19.0–54.2%, respectively; Cd, Cr, and Pb in rice were decreased by 85.9–91.5%, 40.0–76.5%, and 16.4–45.4%, respectively; and these were followed by slightly higher efficacy of L + C + S than L + C. The differences between L + C and L + C + S mainly lie in soil microbial communities, enzymes, and fertility. In YC, treatment with L + C + S increased microbial carbon and activities of urease (EC3.5.1.5) and phosphatase (EC3.1.3.1) by 21.0%, 85.5%, and 22.3%; while treatment with L + C decreased microbial carbon and activities of phosphatase and sucrose (EC3.2.1.26) by 1.31%, 34.9%, and 43.4%, respectively. Moreover, the treatment of FY soils with L + C + S increased microbial carbon and activities of urease, phosphatase, and sucrase by 35.4%, 41.6%, 27.9%, and 7.37%; and L + C treatment only increased the microbial carbon and the activity of phosphatase by 3.14% and 30.3%, respectively. Furthermore, the organic matter and available nitrogen were also increased by 8.8–19.0% and 7.4–14.6% with L + C + S treatments, respectively. These suggested that the combination of L + C + S stimulated the growth of soil microbial communities and increased the activity of soil enzymes. Therefore, the L + C + S strategy can be a practical and effective measure for safe rice production as it was more suitable for the remediation of heavy metals in our experimental sites.

## 1. Introduction

Polluted land is a global issue, especially in developing countries. In recent years, many studies have focused on soil polluted with heavy metals and its negative influence on plants, other living organisms, and even on the human body through the food chain [1,2,3,4,5]. In China, a national soil survey showed that 16.1% of agricultural land was polluted with respect to the national standards of the Ministry of Environmental Protection [6].

Passivators include calcium–silicon materials, carbon materials, and clay minerals, including corresponding nanomaterials, which have been reported in recent years because they allow the in situ remediation of paddy soil contaminated with heavy metals [7,8,9]. Among these passivators, liming materials are widely used because of their low cost and their significant ability to increase the soil pH and decrease the bioavailability of heavy metals in polluted soil [10,11,12]. Materials containing lime are effective passivators for the remediation of soil polluted with heavy metals and they have been shown to significantly decrease grain Cd concentrations under field conditions [11,13,14]. Other passivators, such as biochar and clay minerals, and organic passivators (manure, peat, and bark) used as neutralizing substances [15] can also be researched because of their large surface and eco-friendly properties. The addition of biochar to polluted soil can immobilize heavy metals in soil, reduce their absorption by crops, and improve soil quality [16,17,18]. Neutralizing additives applied in the experiment reduced arsenic concentration in plants with the reducing effect achieved with silt and lime for maize and with compost, lime, and zeolite for orchard grass [19]. Clay minerals, including sepiolite, are also effective in immobilizing heavy metals by increasing the pH of the soil [20,21].

However, the use of passivators for in situ remediation of polluted paddy soil is in the early stages of development; the long-term influence of these passivators on soil and even on the ecological environment is unknown. Although passivators including lime, biochar, clay minerals, fertilizers, and some soil conditioners significantly reduce the bioavailability of heavy metals in soil and their accumulation in plants, their drawbacks cannot be ignored [22,23]. The inappropriate application of these passivators may cause soil problems, such as excess or continuous addition of lime, which may destroy the structure of the soil aggregate, harden the soil, disturb the microbial communities, and reduce the farmland tilling ability [24]. The optimal application of passivators is critical to maximize the utilization efficiency and complement their disadvantages. In addition, a suitable passivator can provide low-cost and eco-friendly techniques and contribute to green sustainable development [25].

In this study, we mixed lime, biochar, and sepiolite in two patterns, which were proven individually effective in previous research, and focused on a typical contaminated site, viz. Shaoxing city in Zhejiang province in South China. We focused on the concentration of heavy metals in rice grain with mixed passivating agents, the bioavailability of heavy metals in soil, and the biological characteristics of soil, which were reflected by microbial carbon, soil enzyme, and fertility levels. The main objective was to provide useful information for optimal passivators in moderately polluted fields and find more suitable mixed passivators that can reduce heavy metals in plants without destroying the soil ecology. Moreover, this study can provide practical and effective measures for safe rice production, especially in view of the confusing use of passivators and their varying effects in agricultural production at this stage.

## 2. Materials and Methods

### 2.1. Experiment Site and Soil Characteristics

The experiment was conducted in Yuecheng (YC), Zhuji (ZJ), and Fuyang (FY), which are located in the suburban area of Zhejiang province, the main rice production region in southern China. The basic properties of the soil are shown in Table 1. The soil from all three sites was acidic, and Cd was the main pollutant, followed by Cr and Pb, which was present at low concentrations. The paddy soil in ZJ, YC, and FY were defined as clay loam with clay, silty clay loam, and silty clay loam, respectively.

### 2.2. Passivator Treatments, Sampling, and Measurements

The experiment was conducted at the three sites simultaneously from June to November in 2019. All treatments included the control with no passivators (CK), lime + biochar (L + C) 9000 kg ha^−1^ at a ratio of 1:1, and lime + biochar + sepiolite (L + C + S) 9000 kg ha^−1^ at a ratio of 1:1:1. Three repeats were conducted at each location in an area of 20 m × 50 m for each treatment as well as randomly arranged plots in the field. The rice cultivar was YY15 (Yongyou15, Indica-Japonica cross three-line hybrid rice, Ningbo Academy of Agricultural Sciences, Ningbo, China), a local, recognized low-Cd-accumulator, which was used at three sites. Approximately 50-day rice seedlings were planted in a field supplied with a passivator 20 days prior, followed by local water and fertilizer management habits until harvest and sampling. Commercial compound fertilizer (compound fertilizer, 15-15-15, produced by China Agro Group Holdings Co., Beijing, China) was applied with a dosage of 60 kg N ha^−1^, 60 kg P_2_O_5_ ha^−1^, and 60 kg K_2_O ha^−1^. During the growing period, urea was applied as the top dressing at tilling, booting, and filling–maturity stages with dosages of 50 kg, 50 kg, and 40 kg N ha^−1^. Water management followed rules of alternating wetting and drying, with flooding, drainage, and intermittent flooding during the stages of seedling, tilling, booting, and filling–maturity during the entire rice growing season. All fertilizers were purchased from local agricultural stores. The mixed passivators included lime (chemical CaO, produced by Hongwei Calcium Powder Factory, Datong Town, Jiande, China), biochar (made from wheat straw, produced by Henan Shangqiu Sanli New Energy Co., Shangqiu, China), and sepiolite (hydrated magnesium silicate, Mg_4_Si_6_O_15_(OH)_2_·6H_2_O, produced by Hebei Lingshou County Baifeng Mineral Products Processing Plant, Shijiazhuang, China). The basic properties of the soil are shown in Table 1.

During harvest, the soil of each treatment was collected. The physicochemical parameters, microbial carbon, and available Cd, Cr, and Pb were determined to evaluate the efficacy of the passivators. The soil samples were naturally air dried and then ground through a 2 mm sieve for determination of physicochemical parameters, including pH, organic matter (OM), available nitrogen (AN), available phosphorus (AP), available potassium (AK), diethylenetriamine penta acetic acid (DTPA)-extractable Cd (DTPA-Cd), DTPA-extractable Cr (DTPA-Cr), and DTPA-extractable Pb (DTPA-Pb). The fresh soils, after being freeze dried and coarsely ground, were used to determine microbial carbon and soil enzymes. The microbial carbon was determined via chloroform fumigation; the soil activities of sucrase (EC3.2.1.26), phosphatase (EC3.1.3.1), and urease (EC3.5.1.5) were determined via enzymatic reactions. Soil pH (pH) was determined potentiometrically in distilled water at the ratio of 1:2.5 (*w*/*v*) using a pH meter (PHS-3C, Shanghai Yidian Scientific Instruments Co., Shanghai, China) and pH glass electrode (E-201-C, Shanghai Yidian Scientific Instruments Co., Shanghai, China). The OM was determined via external potassium dichromate oxidation and the titration method [26]. The cation exchange capacity (CEC) was determined via neutral ammonium acetate extraction, boric acid absorption, and titration methods. The AN was determined via the micro-Kjeldahl method. The AP was extracted with NH_4_F-HCl solution and determined by the formation of the blue phosphomolybdate complex via spectrophotometry (UV-6000, Shanghai Yuan analysis Instruments Co., Shanghai, China), and the available potassium (AK) was extracted by ammonium acetate and determined using an aflame photometer (6400A, Shanghai Precision Scientific Instruments Co., Shanghai, China). The DTPA-Cd, DTPA-Cr, and DTPA-Pb were determined by inductively coupled plasma–mass spectrometry (iCAP Q™ ICP-MS, Thermo Fisher Corporation, Madison, WI, USA). The background parameters of soil were determined in the same way as the samples, except that soil clay, silt, and sand were determined by a specific gravimetric method using a soil gravimeter (TM-85, Hebei Wuqiang County Tonghui Instrument Factory, Hengshui, China). During harvest, the rice grains were sampled in the field and brought to the laboratory, where they were dried in an oven at 80 °C for over 5 h. The grain was shelled to rice, crushed into powder, and stored at room temperature for testing. The Cd, Cr, and Pb contents in rice were determined via inductively coupled plasma–mass spectrometry (iCAP Q™ ICP-MS, Thermo Fisher Co., Madison, WI, USA).

Two standard materials (rice GBW10045 and soil GBW07415a, Chemical Metrology & Analytical Science Division, National Institute of Metrology, Beijing, China), purchased from the National Research Center for Standards in China, were used to control the analysis quality of grain and soil heavy metals. The recovery efficiency for rice and soil standard materials was 96.2% and 94.9%, respectively.

### 2.3. Statistical Analysis

Statistical analysis of the experimental data was performed with Microsoft^®^ Excel 2010 (Microsoft Corporation, Albuquerque, NM, USA) and SPSS 19.0 software (SPSS Inc., Chicago, IL, USA), with significant differences determined by one-way analysis of variance and Duncan’s multiple range tests for three locations separately.

## 3. Results and Discussion

### 3.1. Grain Heavy Metals (Cd, Cr, and Pb)

**Figure 1:**
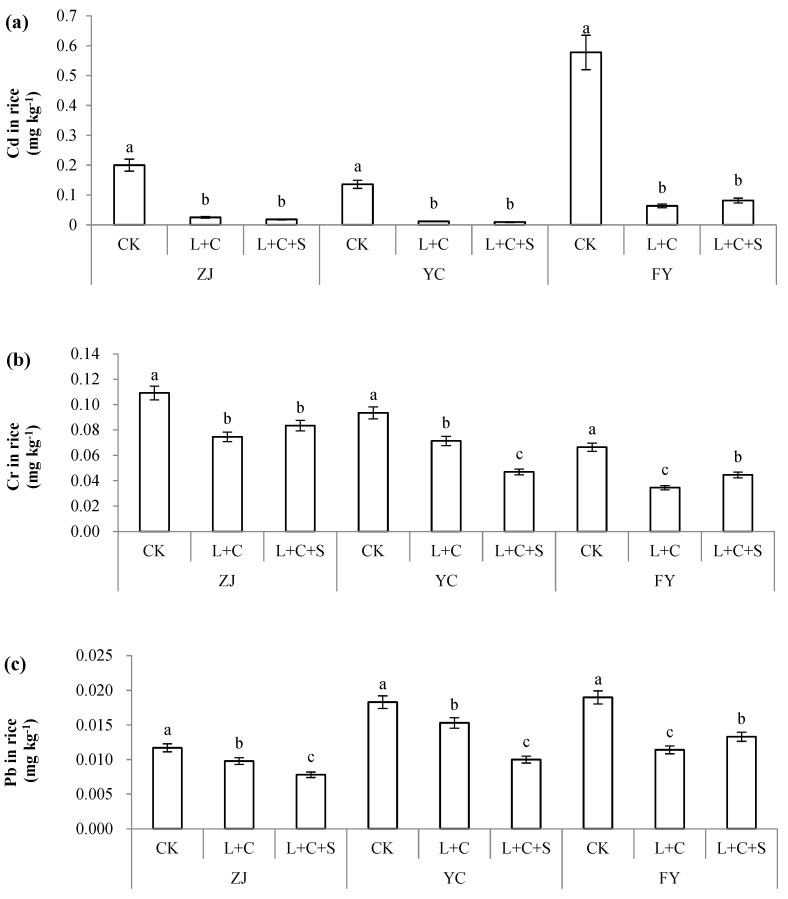
Cd (**a**), Cr (**b**), and Pb (**c**) in rice treated by control with no passivators (CK), 9000 kg ha^−1^ of lime + biochar with a rate of 1:1 (L + C), and 9000 kg ha^−1^ lime + biochar + sepiolite with a rate of 1:1 (L + C + S) in paddy soil. Values (means ± SD, *n* = 3) with different letters indicate significant differences between objects in the respective sites (separately for ZJ (Zhuji), YC (Yuecheng), or FY (Fuyang)).

Treated with passivators in soil, including L + C or L + C + S, the contents of Cd, Cr, and Pb in rice decreased significantly at the three field sites (Figure 1 and Table 1). Compared with the CK treatments, L + C decreased Cd in rice by 87.6%, 91.5%, and 89.0%, and the treatment with L + C + S decreased Cd in rice by 91.0%, 93.2%, and 85.9% in ZJ, YC, and FY, respectively. The Cr content in rice decreased by 46.4%, 53.6%, and 76.5% when treated with L + C, and by 40.0%, 69.4%, and 69.6% when treated with L + C + S in ZJ, YC, and FY, respectively. Furthermore, Pb in rice decreased by 16.4%, 16.4%, and 40.0% when treated with L + C, and by 33.3%, 45.4%, and 30.0% when treated with L + C + S in ZJ, YC, and FY, respectively (Figure 1).

According to the efficacy in reducing heavy metals in rice, treatment with L + C and L + C + S decreased the amounts of heavy metals (Cd, Cr, and Pb) by 40.0–89.0% and 30.0–85.9% in FY, 16.4–91.5% and 45.4–93.2% in YC, and 16.4–87.6% and 33.3–91.0% in ZJ, respectively. All data showed that L + C treatment had slightly higher efficacy than L + C + S in FY (89.0 to 85.9%, 76.5 to 59.6%, and 40.0 to 30.0% for Cd, Cr, and Pb, respectively), whereas the efficacy of the L + C + S treatment was slightly higher than that of L + C in YC (93.2 to 91.5%, 69.4 to 53.6%, and 45.4 to 16.4% for Cd, Cr, and Pb, respectively). Simultaneously, we noticed lower values of pH, OM, AN, AP, and CEC in FY than in YC. Furthermore, the efficacy of the L + C + S treatment in reducing Cd and Pb in rice was higher than that of L + C at the ZJ site (91.0% to 87.6% for Cd and 33.3% to 16.4% for Pb), whose background values of soil properties were similar to those in YC (Figure 1). Thus, the efficacy of passivators in reducing heavy metals in rice varied because of the different physicochemical properties of the soil.

### 3.2. Soil pH

**Figure 2:**
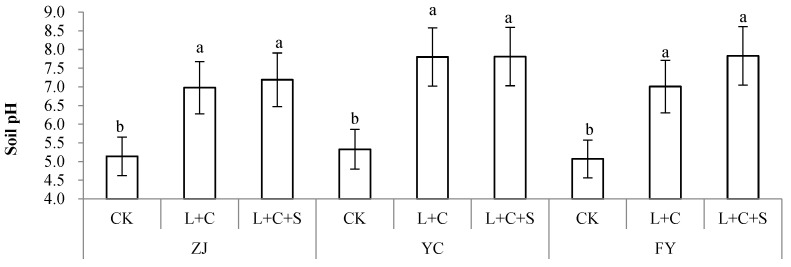
Paddy soil pH treated by control with no passivators (CK), 9000 kg ha^−1^ of lime + biochar with a rate of 1:1 (L + C), and 9000 kg ha^−1^ lime + biochar + sepiolite with a rate of 1:1 (L + C + S). Values (means ± SD, *n* = 3) with different letters indicate significant differences between objects in the respective sites (separately for ZJ (Zhuji), YC (Yuecheng), or FY (Fuyang)).

As shown in Figure 2, the treatments with L + C and L + C + S increased the soil pH significantly at ZJ, YC, and FY. The L + C treatments increased the soil pH by 1.84, 2.47, and 1.94 units, whereas the L + C + S treatments increased it by 2.08, 2.48, and 2.76 units at the ZJ, YC, and FY sites, respectively. Overall, the mixture of L + C + S improves the soil pH more than L + C.

### 3.3. DTPA-Extractable Heavy Metals (Cd, Cr, and Pb)

**Figure 3:**
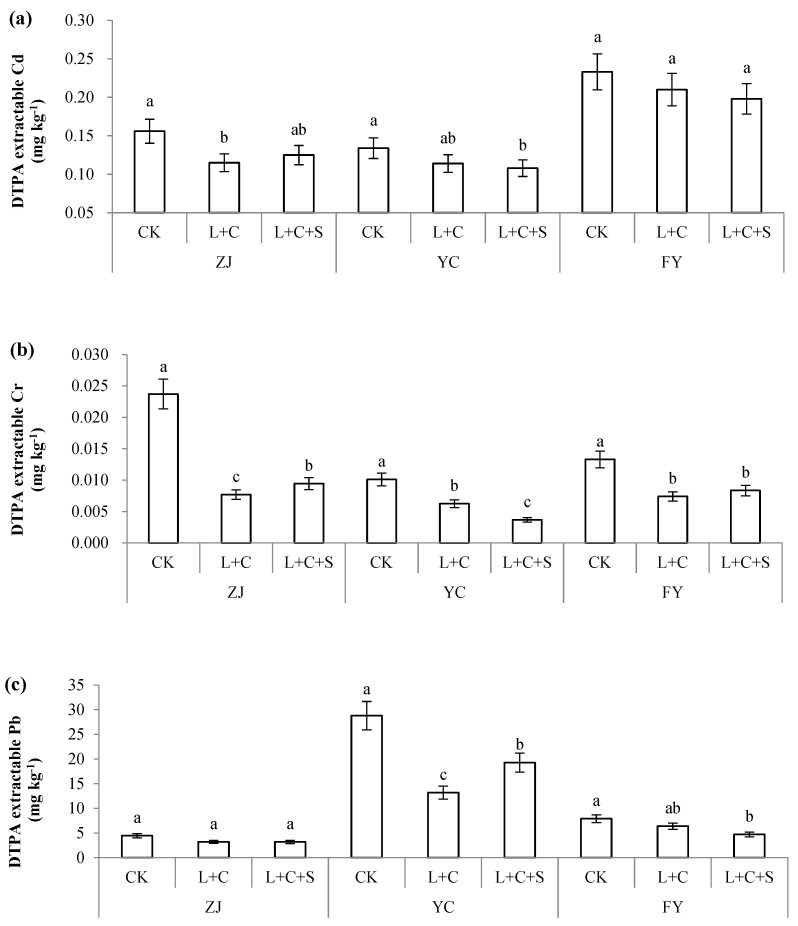
DTPA-extractable Cd (**a**), Cr (**b**), and Pb (**c**) in paddy soil treated by control with no passivators (CK), 9000 kg ha^−1^ of lime + biochar with a rate of 1:1 (L + C), and 9000 kg ha^−1^ lime + biochar + sepiolite with a rate of 1:1 (L + C + S). Values (means ± SD, *n* = 3) with different letters indicate significant differences between objects in the respective sites (separately for ZJ (Zhuji), YC (Yuecheng), or FY (Fuyang)).

The direct result of increased soil pH was the reduction of DTPA-extractable heavy metals. We mainly focused on the bioavailable Cd, Cr, and Pb, which are the primary metal pollutants in the experimental sites. Besides the significantly increased soil pH and decreased Cd, Cr, and Pb contents in rice, the DTPA-extractable heavy metals in soil decreased (Figure 3).

Soil DTPA-Cd decreased by 9.87–26.3% under treatment with the passivators in the three plot sites (Figure 3a). Under the L + C and L + C + S treatments, the soil DTPA-Cd decreased by 26.3% and 19.9% in ZJ, 14.9% and 19.4% in YC, and 9.87% and 15.0% in FY, respectively. Although both treatments decreased DTPA-Cd in soil, a significant difference occurred only in YC with L + C + S and ZJ with L + C. Soil DTPA-Cr decreased by 37.2–67.5% when treated with passivators in three field sites (Figure 3b). The DTPA-Cr was decreased by 67.5%, 38.1%, and 44.4% when treated with L + C, whereas it decreased by 60.1%, 63.6%, and 37.2% when treated with L + S + C in ZJ, YC, and FY, respectively. Compared with the CK treatment, L + C or L + C + S showed significantly lower DTPA-Cr at all three sites (Figure 3b). Soil DTPA-Pb decreased by 19.0–54.2% when treated with passivators in three field sites (Figure 3c). A significant decrease occurred in YC (L + C and L + C + S) and FY (L + C + S). DTPA-Pb decreased by 28.0%, 54.2%, and 19.0% with L + C, and 28.5%, 33.0%, and 40.3% with L + C + S in ZJ, YC, and FY, respectively.

According to the variation of bioavailable heavy metals in soil, several agronomic practices to improve soil quality in heavy-metals-polluted soils, including soil remediation [27], fertilizer treatments [28], irrigation management [29,30,31], and tillage strategies [32]. Biochar can immobilize heavy metals in polluted soil, thereby reducing its bioavailability and accumulation in agro products [33]. The incubation experiment showed that multiple-modified biochar could immobilize Cd in polluted soil by transforming it into a more stable state and decreasing DTPA-Cd by 92.02% for farmland soil and 90.27% for vegetable soil [34]. Besides their direct influence on soil metals, passivators such as sepiolite reduced Cd in rice grain by 47–49% [21]. The application of lime in the soil also decreased the concentration of Cd in plants by 20–37.5% [35]. However, although grain Cd in severely polluted acidic soil decreased significantly by the application of lime, it remained higher than the limiting value of China (0.2 mg kg^−1^), even when assisted by water management [23]. Our results confirmed that the addition of mixed passivators in polluted fields increased the soil pH and decreased grain metals and soil bioavailable metals, and also revealed the different efficacies of L + C and L + C + S treatments in immobilizing heavy metals among experimental sites or different heavy metals, although DTPA-Cd, DTPA-Cr, and DTPA-Pb were all decreased by both L + C and L + C + S (Figure 3). The L + C + S treatment exhibited a larger decrease step in DTPA-Cd than those of L + C in YC (19.4% and 14.9%) and FY (15.0% and 9.87%), respectively, which had higher background total Cd in soil than ZJ despite their different soil properties (Figure 3a). For DTPA-Cr, a similar larger decrease step with L + C + S than L + C occurred only in YC (63.6 to 38.1%), where the background total Cr was the highest among the three sites (Figure 3b). For DTPA-Pb, a larger decrease step of L + C + S than those of L + C only occurred in FY (40.3 to 19.0%), where the total background Pb was 80.20 mg kg^−1^, in contrast to that in YC (33.0% and 54.2%), which showed a total background Pb of 78.90 mg kg^−1^ (Figure 3c). Therefore, in our experiment, it seems the efficacy of passivators in reducing soil DTPA-Cd or DTPA-Cr was higher with L + C + S than L + C treatment when the background values of total Cd or Cr were higher than some threshold values. Correlation analysis found the DTPA-Cd was significantly negatively related to soil fertilizer parameters, including OM, AN, AP, and CEC, with the correlation coefficient of −0.945, −0.821, −0.721, and −0.741, respectively (Table 2). We can also see from Figure 3a, the soil DTPA-Cd content of ZJ or YC with higher fertility levels was generally lower than that of FY soil with lower fertility levels; also, in terms of the percentage of DTPA-Cd to total Cd, it was lower in ZJ and YC soil than in FY soil (43.1, 33.1 and 48.5%). Therefore, a high fertility level favors suppression of soil Cd activation, while the soil DTPA-Cr only exhibited significantly negative results related to soil pH value (*r* = −0.763). However, it is different for DTPA-Pb, whose reduction may be more likely to be influenced by the background Pb and properties of the soil, including pH, OM, AN, AP, CEC, and other unknown factors. Correlation analysis revealed that soil DTPA-Pb was positively related to soil AN and CEC, with correlation coefficients of 0.674 and 0.853, respectively (Table 2). In Figure 3c, the DTPA-Pb content of YC soil was higher than those of FY soil with comparable total Pb levels between the two sites and higher AN and CEC in the YC site. Moreover, in terms of the proportion of DTPA-Pb to total Pb, the YC soil was higher than the FY soil (35.7% and 9.85%). A review showed that the efficacy of biochar in immobilizing heavy metals depends on many factors in the field, including site climate, agronomic measures, biochar dosage, feedstock, and properties [16]. Furthermore, the soil background physical properties such as pH, clay, and OM content, as well as its biochemical properties such as the microbial community and enzymes related to nutrient transformation, were all critical for the efficacy of passivators in immobilizing heavy metals in soils [25]. The interaction between different passivators and soil physicochemical indicators is a complex process worth studying. Based on this complex relationship, the different efficacies between L + C and L + C + S did not show a clear pattern influenced by physicochemical properties of the soil (Figure 3). Meanwhile, correlation analysis not only confirmed the positive correlation between rice heavy metal content and soil DTPA extractable metals, but also found a significant positive correlation between rice Cd and soil AK (*r* = 0.879) and a significant negative correlation between rice Pb and soil AP (*r* = −0.668), which, of course, also included the contribution of metal uptake and transport efficiency of rice (Table 2). In a word, all these significantly or weakly decreased DTPA-Cd, DTPA-Cr, and DTPA-Pb with the passivator treatments in our results, which suggested that the efficacy of different mixed passivators relies on multiple factors, including heavy metals, soil properties, and even climatic conditions and agronomic measures among experiment sites.

### 3.4. Microbial Carbon and Soil Enzymes

**Figure 4:**
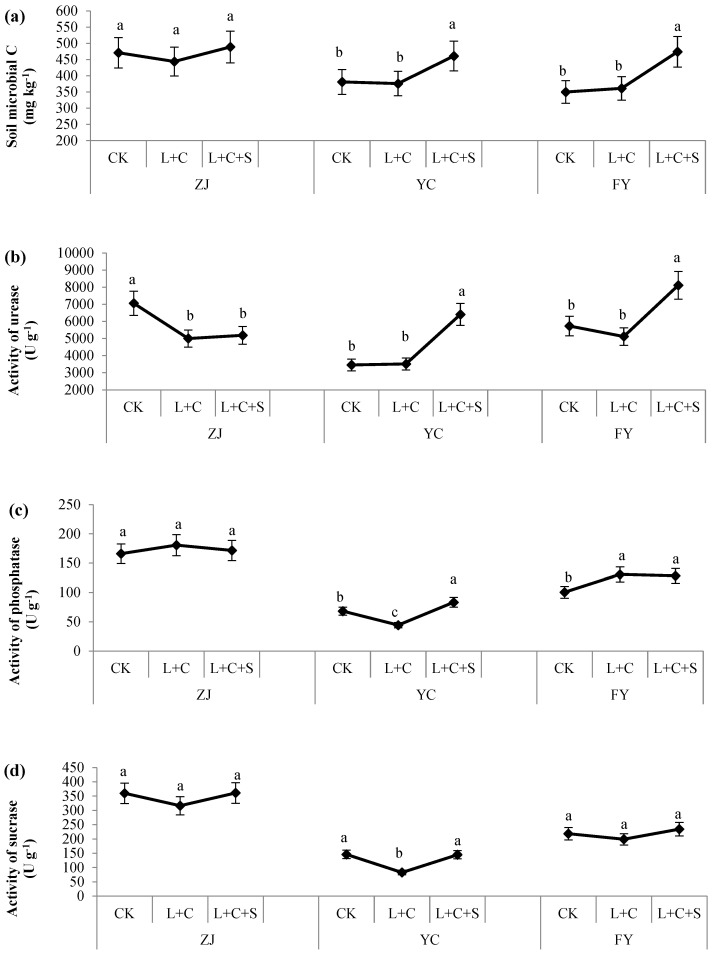
Soil microbial carbon (**a**) and activities of urease (**b**), phosphatase (**c**), and sucrose (**d**) in paddy soil treated by control with no passivators (CK), 9000 kg ha^−1^ of lime + biochar with a rate of 1:1 (L + C), and 9000 kg ha^−1^ lime + biochar + sepiolite with a rate of 1:1 (L + C + S). Values (means ± SD, *n* = 3) with different letters indicate significant differences between objects in the respective sites (separately for ZJ (Zhuji), YC (Yuecheng), or FY (Fuyang)).

Many studies have focused on the efficacy of passivators on rice heavy metals for the safety of agro products or focused on bioavailable heavy metals for the remediation of polluted soil. Some research has focused on the physicochemical properties of soil only aiming to improve the efficacy of passivators in reducing heavy metals in agro products or polluted soil. To date, little is known about the drawbacks or ecological properties of soil resulting from the addition of various passivators. Passivators such as biochar, clay minerals, and lime are commonly used in polluted soils to immobilize heavy metals by various mechanisms, including complexation, precipitation, ion exchange, electrostatic interaction, and redox reactions depending on the soil pH, amount and type of minerals, redox potential, sorption capacity, and organic matter content [12,25,36,37]. During the interaction between passivators and soil, the soil properties, including pH, OM, and redox potential, are critical for the efficacy of passivators in heavy metal immobilization [25,38]. Meanwhile, it was neglected that the soil quality including ecological and physicochemical properties were all influenced by the passivators as showed in our research. We measured the soil microbial carbon and the activities of urease, phosphatase, and sucrose treated by L + C, L + C + S, and CK (Figure 4). Compared with the CK treatment, L + C + S significantly enhanced the microbial carbon and the activities of urease and phosphatase in YC (by 21.0%, 85.5%, and 22.3%, respectively) and FY (by 35.4%, 41.6%, and 27.9%, respectively), whereas the activity of urease decreased significantly in ZJ (by 26.5%). Meanwhile, there was no significant variation in microbial carbon, the activities of phosphatase and sucrose at ZJ, as well as that of sucrose in YC or FY treated with L + C + S compared with those with the CK treatment. Furthermore, compared with the CK treatment, although L + C in soil significantly increased the activity of phosphatase in FY (by 30.3%), which was the only significant positive result of the L + C treatment, L + C also significantly decreased the activities of urease in ZJ (by 29.2%), phosphatase (by 34.9%), and sucrose (by 43.4%) in YC. Meanwhile, there were no significant differences between CK and L + C in microbial carbon in ZJ, YC, or FY, the activities of urease in YC and FY, the activities of phosphatase in ZJ, and the activities of sucrose in ZJ and FY. These results suggest that the combination of L + C + S stimulates the increase in or enrichment of the soil microbial community and accelerates the activity of soil enzymes related to the conversion of nitrogen and phosphorus. Furthermore, L + C in paddy soil did not significantly increase the microbial carbon and enzymes in the soil, except for phosphatase in FY (Figure 4). Comprehensive comparison showed that L + C + S was more suitable for the remediation of heavy metals with retaining soil ecological properties than L + C in our experimental sites.

In fact, the advantage of mixed passivators has been reported previously. Researchers proved that a mixture of passivators had a superimposed effect on the reduction of bioavailable Cd in soil over any passivators applied individually [39]. Application of biochar + lime is more effective than their individual application in reducing the availability of Pb in soil and its accumulation in rice [40]. An appropriate mixture of passivators decreases bioavailable Cd in soil and grain, as well as increases the diversity and species richness of soil microbial communities [24]. The mixture of sepiolite + organic passivators increased the amounts of AP, AK, OM, microbial carbon, and dehydrogenase activity by 9.6–68.2%, 1.2–28.3%, 37.5–70.5%, 4.1–121.0%, and 6.8–56.8% compared with those of the CK treatment, respectively [41]. Moreover, the mixture of biochar and soda residues was the most effective in reducing Cd and Pb in grain and had the longest-lasting effects with more economic benefit than those of biochar or soda residues individually in acid-polluted soil [42]. However, our data showed this advantage may vary depending on the background properties of the soil. The significant increase in soil microbial carbon and activities of urease or phosphatase were measured with L + C + S treatments at YC and FY sites; while in the ZJ soil, no significant increases were found in microbial carbon and the activities of phosphatase or sucrose, and even significantly decreased activities of urease were detected with L + C + S treatment (Figure 4). Correlation analysis revealed the significant negative relation between DTPA-Pb and soil phosphatase or sucrase, while no significant relation was revealed between DTPA-Cd and any soil enzymes (Table 2). Combined with the contrary effect of AN or CEC to DTPA-Cd (negative correlation) and DTPA-Pb (positive correlation), they may indicate different activation processes of soil Pb and Cd. These results show the complex interaction between passivators and soil and, especially, that the microbial communities that contribute to the enhanced microbial carbon and soil enzymes in L + C + S treatments still need further research.

### 3.5. Physicochemical Characteristics of Soil

Furthermore, besides microbial carbon and soil enzymes, the soil physicochemical properties and fertility varied synchronously as shown in Table 3. The treatment with passivators increased the OM by 6.9–19.0% compared with the CK, except for L + C in YC; this may be attributed to biochar in the passivators (Table 3). However, the different treatments show varied ranges in OM, whose increase step was larger when treated with L + C + S than those with L + C treatment compared with CK in all three sites (Table 3). This may be due to their higher content of soil microbial carbon, as shown in Figure 4. No significant difference was found in CEC except for a slight decrease, which was in most cases supplied by passivators, except for L + C + S in ZJ, which showed a 3.2% increased CEC compared with that of the CK treatment (Table 3). The AN in soil varied depending on the passivators. Treatment with L + C + S increased the AN by 14.6% and 7.4%, whereas treatment with L + C slightly decreased the AN by 5.7% and 8.4% in YC and FY, respectively; this was in accordance with the activity of urease (Figure 4). In ZJ, with significantly decreased activities of urease under the L + C and L + C + S treatments (Figure 4), the AN was synchronously decreased by 4.4% and 3.3%, respectively (Table 3). The AP was increased by the L + C and L + C + S treatments in ZJ (by 79.1% and 38.4%) and FY (by 26.5% and 43.4%) significantly (Table 3), and also increased by 6.9% and 27.2% in YC, respectively. Treated with L + C and L + C + S, the AK was significantly decreased in FY, while slightly increased by 8.7% and 2.4% in ZJ, and then slightly decreased by 11.9% and 2.0% in YC, respectively.

Previous research shows that the application of passivators in polluted soil improves the physicochemical and biological properties of the soil, including the enzyme activity and microbial diversity, and it promotes crop growth [17,38,43,44,45,46]. Moreover, the application of multiple-modified biochar significantly increased soil dehydrogenase, OM, and AK [34]. Our results show that the OM was increased by 8.8–19.0% and 6.9–13.2%; the AP was increased by 27.2–43.4% and 6.9–79.1% with the L + C + S and L + C treatments, respectively (Table 3). While the AN was increased by 7.4–14.6% with the L + C + S treatment, no increase was found with the L + C treatment. The variation of soil fertility resulting from the addition of passivators suggested again that the mixture of L + C + S may be more suitable for contaminated soil remediation than L + C for eco-friendly standards, while the stability of L + C + S among different sites may be critical in future practice. Furthermore, one of the deficiencies in our research is that we did not know the microbial species stimulated by the passivators, for example, the addition of biochar to As-polluted soil increased the activities of soil enzymes and the richness of firmicutes and proteobacteria while decreasing the richness of Bacteroidetes [47]. Meanwhile, the response of available nutrient elements and activities of their related enzymes to passivators were not perfectly synchronized or weakly correlated (Figure 4 and Table 3). This asynchronous response may be related to the different microbial species increased in the soil caused by passivator application. In a word, the interaction between passivators and soil is a complex process, which still needs further research together with a study of the influence of agronomic practices. Then, stable, long term and effective passivators based on soil eco-friendly standards can be established. Because of its ability to improve soil microbial carbon, soil enzyme activity, and soil fertility (Figure 4 and Table 3), the L + C + S measure possesses a more practical application value for moderately or lightly polluted farmland, especially for arable land with degraded quality.

## 4. Conclusions

In this study, different mixtures of passivators, i.e., L + C or L + C + S, significantly decreased the amounts of Cd, Cr, and Pb in rice, increased the soil pH by 1.84–2.76 units, and decreased the soil DTPA-Cd, Cr, and Pb by 9.87–26.3%, 37.2–67.5%, and 19.0–54.2%, respectively. Differences between L + C and L + C + S were mainly found in soil microbial communities, enzymes, and fertility. The significantly higher microbial carbon and higher activities of urease and phosphatase with L + C + S treatment in YC and FY suggested that the L + C + S stimulated the growth of soil microbial communities, especially some microbes related to soil enzymes which are responsible for the conversion of nitrogen and phosphorus in soil. For soil fertilizer parameters, the OM was increased by 6.9–19.0% followed by a larger increase step with the L + C + S than L + C treatments. Treatment of L + C + S increased AN by 14.6% and 7.4%, while L + C decreased AN by 5.7% and 8.4% in YC and FY, respectively. All data indicated that a mixture of L + C + S, as a practical and effective measure, is more suitable for the remediation of heavy metals in our experimental sites. The results of this study provide an effective and practical technique for heavy metal passivation on moderately or lightly contaminated rice fields by comparing the results of three site experiments. Future research will deeply investigate more economical and practical measures, especially for degraded arable land and their stability in different sites, and also examine concerns about related mechanisms between passivators and soil interaction.

## Figures and Tables

**Table 1:** Basic properties of soil, lime, sepiolite, and biochar.

Parameters	ZJ	YC	FY	Lime (L)	Sepiolite (S)	Biochar (C)
Organic matter (g kg^−1^)	35.4	47.5	27.4	/	/	/
Available nitrogen (mg kg^−1^)	169	184	135	/	/	/
Available phosphorus (mg kg^−1^)	38.9	36.2	11.3	/	/	/
Cation exchange capacity (cmol (+) kg^−1^)	11.8	17.5	9.9	/	/	/
Clay (%)	29	32	28	/	/	/
Silt (%)	33	58	60	/	/	/
Sand (%)	38	10	12	/	/	/
pH	5.33	5.48	4.94	12.5	9.50	9.95
Cd (mg kg^−1^)	0.362	0.405	0.481	0.113	0.137	0.195
Cr (mg kg^−1^)	28.2	62.1	47.5	8.51	7.85	16.1
Pb (mg kg^−1^)	45.6	78.9	80.2	10.4	9.51	7.3

**Table 2:** Correlation coefficient of soil physicochemical parameters and Cd, Cr, and Pb in rice.

Parameters	pH	DTPA−Cd	DTPA−Cr	DTPA−Pb	MC	UA	PA	SA	OM	AN	AP	AK	CEC	Rice Cd	Rice Cr	Rice Pb
pH	1	−0.354	−0.763 *	−0.147	0.325	0.094	−0.083	−0.259	0.269	0.166	0.217	−0.417	0.065	−0.743 *	−0.626	−0.532
DTPA−Cd	−0.354	1	0.282	−0.326	−0.417	0.384	0.077	0.057	−0.945 **	−0.821 **	−0.721 *	0.690 *	−0.741 *	0.689 *	−0.364	0.419
DTPA−Cr	−0.763 *	0.282	1	−0.281	0.134	0.305	0.387	0.573	−0.337	−0.220	−0.076	0.047	−0.264	0.499	0.696 *	0.150
DTPA−Pb	−0.147	−0.326	−0.281	1	−0.345	−0.486	−0.768 *	−0.707 *	0.588	0.674 *	−0.259	0.037	0.835 **	−0.058	0.095	0.496
MC	0.325	−0.417	0.134	−0.345	1	0.589	0.581	0.616	0.263	0.289	0.575	−0.511	−0.077	−0.456	0.195	−0.727 *
UA	0.094	0.384	0.305	−0.486	0.589	1	0.441	0.434	−0.455	−0.222	−0.129	0.127	−0.589	0.140	−0.238	−0.298
PA	−0.083	0.077	0.387	−0.768 *	0.581	0.441	1	0.950 **	−0.327	−0.418	0.568	−0.241	−0.654	−0.082	0.188	−0.662
SA	−0.259	0.057	0.573	−0.707 *	0.616	0.434	0.950 **	1	−0.273	−0.334	0.529	−0.183	−0.561	0.064	0.418	−0.545
OM	0.269	−0.945 **	−0.337	0.588	0.263	−0.455	−0.327	−0.273	1	0.932 **	0.502	−0.538	0.893 **	−0.573	0.319	−0.220
AN	0.166	−0.821 **	−0.220	0.674 *	0.289	−0.222	−0.418	−0.334	0.932 **	1	0.312	−0.383	0.880 **	−0.406	0.286	−0.096
AP	0.217	−0.721 *	−0.076	−0.259	0.575	−0.129	0.568	0.529	0.502	0.312	1	−0.551	0.127	−0.507	0.367	−0.668 *
AK	−0.417	0.690 *	0.047	0.037	−0.511	0.127	−0.241	−0.183	−0.538	−0.383	−0.551	1	−0.343	0.879 **	−0.241	0.649
CEC	0.065	−0.741 *	−0.264	0.835 **	−0.077	−0.589	−0.654	−0.561	0.893 **	0.880 **	0.127	−0.343	1	−0.356	0.335	0.202
Rice Cd	−0.743 *	0.689 *	0.499	−0.058	−0.456	0.140	−0.082	0.064	−0.573	−0.406	−0.507	0.879 **	−0.356	1	0.155	0.662
Rice Cr	−0.626	−0.364	0.696 *	0.095	0.195	−0.238	0.188	0.418	0.319	0.286	0.367	−0.241	0.335	0.155	1	0.118
Rice Pb	−0.532	0.419	0.150	0.496	−0.727 *	−0.298	−0.662	−0.545	−0.220	−0.096	−0.668 *	0.649	0.202	0.662	0.118	1

Notes: “*” means significant level at 0.05, “**” means significant level at 0.01. The polluted soil at the ZJ (Zhuji), YC (Yuecheng), or FY (Fuyang) sites was treated by control with no passivators (CK), 9000 kg ha^−1^ of lime + biochar with a rate of 1:1 (L + C), and 9000 kg ha^−1^ lime + biochar + sepiolite with a rate of 1:1 (L + C + S). During harvest, the soil and rice of each treatment was collected, and the soil physicochemical parameters, soil microbial carbon (MC), soil urease activity (UA), phosphatase activity (PA), sucrose activity (SA), and Cd, Cr, and Pb in rice were determined. Correlation analyses were made by SPSS 19.0 (*n* = 9).

**Table 3:** Physicochemical properties of paddy soil.

**Experiment plot**	**Treatments**	**OM**			**CEC**		
		**Values, g kg** **^−1^**	**Increase/** **Decrease, %**		**Values, cmol** **(+) kg** **^−1^**		**Increase/** **Decrease, %**
ZJ	CK	38.5 ± 3.0	/		12.4 ± 0.9		/
	L + C	43.6 ± 4.1	+13.2		12.0 ± 1.0		−3.2
	L + C + S	45.8 ± 3.5	+19.0		12.8 ± 1.0		+3.2
YC	CK	50.2 ± 4.9	/		19.1 ± 1.2		/
	L + C	49.5 ± 3.8	−1.4		17.7 ± 1.1		−7.3
	L + C + S	54.6 ± 5.1	+8.8		17 ± 1.1		−11.0
FY	CK	27.4 ± 2.6	/		10.0 ± 0.9		/
	L + C	29.3 ± 3.0	+6.9		9.7 ± 0.9		−3.0
	L + C + S	30.5 ± 3.5	+11.3		9.9 ± 0.8		−1.0
**Experiment plot**	**Treatments**	**A** **N%**		**A** **P%**		**A** **K%**	
		**Values,** **mg kg^−1^**	**Increase/** **Decrease, %**	**Values,** **mg kg^−1^**	**Increase/** **Decrease, %**	**Values,** **mg kg^−1^**	**Increase/** **Decrease, %**
ZJ	CK	172.8 ± 15.1	/	25.8 ± 3.2	/	73.6 ± 8.2	/
	L + C	165.2 ± 10.9	−4.4	46.2 ± 3.6	+79.1	80.0 ± 9.5	+8.7
	L + C + S	167.1 ± 12.3	−3.3	35.7 ± 3.5	+38.4	75.4 ± 9.3	+2.4
YC	CK	198.9 ± 15.9	/	20.2 ± 2.8	/	86.8 ± 7.9	/
	L + C	187.5 ± 16.3	−5.7	21.6 ± 3.7	+6.9	76.5 ± 9.3	−11.9
	L + C + S	227.9 ± 18.5	+14.6	25.7 ± 3.1	+27.2	85.1 ± 8.6	−2.0
FY	CK	135.1 ± 11.9	/	11.3 ± 0.8	/	131.0 ± 10.5	/
	L + C	123.7 ± 13.6	−8.4	14.3 ± 1.3	+26.5	83.6 ± 9.1	−36.2
	L + C + S	145.1 ± 10.8	+7.4	16.2 ± 1.4	+43.4	90.8 ± 8.7	−30.7

Notes: The polluted soils at the ZJ (Zhuji), YC (Yuecheng), or FY (Fuyang) sites was treated by control with no passivators (CK), 9000 kg ha^−1^ of lime + biochar with a rate of 1:1 (L + C), and 9000 kg ha^−1^ lime + biochar + sepiolite with a rate of 1:1 (L + C + S). During harvest, the soil of each treatment was collected and the physicochemical parameters were determined. Values were means ± SD, *n* = 3. Decrease was calculated by [(values (L + C/L + C + S)-values (CK))/values (CK)] × 100%.

## Data Availability

The datasets used and analyzed during the current study are available from the corresponding author on reasonable request. All data generated or analyzed during this study are included in this published article.
